# Image cytometry accurately detects DNA ploidy abnormalities and predicts late relapse to high-grade dysplasia and adenocarcinoma in Barrett's oesophagus following photodynamic therapy

**DOI:** 10.1038/sj.bjc.6605688

**Published:** 2010-05-11

**Authors:** J M Dunn, G D Mackenzie, D Oukrif, C A Mosse, M R Banks, S Thorpe, P Sasieni, S G Bown, M R Novelli, P S Rabinovitch, L B Lovat

**Affiliations:** 1 Department of Surgery, National Medical Laser Centre, University College London, 67-73 Riding House Street, London W1W 7EJ, UK; 2 Department of Histopathology, University College London, London, UK; 3 Department of Gastroenterology, University College London Hospitals NHS Trust, London, UK; 4 Cancer Research UK Centre for Epidemiology, Department of Mathematics and Statistics, Wolfson Institute of Preventive Medicine, Bart's & The London School of Medicine, Queen Mary University of London, London, UK; 5 Department of Histopathology, University of Washington (UW), Seattle, WA, USA

**Keywords:** Barrett's oesophagus, oesophageal adenocarcinoma, high-grade dysplasia, DNA ploidy, image cytometry, photodynamic therapy

## Abstract

**Background and aims:**

DNA ploidy abnormalities (aneuploidy/tetraploidy) measured by flow cytometry (FC) are strong predictors of future cancer development in untreated Barrett's oesophagus, independent of histology grade. Image cytometric DNA analysis (ICDA) is an optical technique allowing visualisation of abnormal nuclei that may be undertaken on archival tissue. Our aim was to determine the accuracy of ICDA *vs* FC, and evaluate DNA ploidy as a prognostic biomarker after histologically successful treatment with photodynamic therapy (PDT).

**Methods:**

Nuclei were extracted from 40 *μ*m sections of paraffin-embedded biopsies and processed for ICDA at UCL and FC at UW using standardised protocols. Subsequently, DNA ploidy was evaluated by ICDA on a cohort of 30 patients clear of dysplasia 1 year after aminolaevulinic acid PDT for high-grade dysplasia (HGD). The results were correlated with long-term outcome.

**Results:**

In the comparative study, 93% (41 out of 44) of cases were classified identically. Errors occurred in the near-diploid region by ICDA and the tetraploid region by FC. In the cohort study, there were 13 cases of late relapse (7 cancer, 6 HGD) and 17 patients who remained free of dysplasia after a mean follow-up of 44 months. Aneuploidy post-PDT was highly predictive for recurrent HGD or cancer with a hazard ratio of 8.2 (1.8–37.8) (log-rank *P*=0.001).

**Conclusions:**

ICDA is accurate for the detection of DNA ploidy abnormalities when compared with FC. After histologically successful PDT, patients with residual aneuploidy are significantly more likely to develop HGD or cancer than those who become diploid. DNA ploidy by ICDA is a valuable prognostic biomarker after ablative therapy.

The incidence of oesophageal adenocarcinoma is rising rapidly in the developed world. Barrett's oesophagus (BE) is a precursor lesion that confers an increased risk of oesophageal adenocarcinoma, with incidence rates of 0.4–2% per annum (Cameron *et al*, 1985; Robertson *et al*, 1988; Hameeteman *et al*, 1989; Williamson *et al*, 1991). Progression seems to occur through a metaplasia–dysplasia–carcinoma sequence (Weston *et al*, 1999; Montgomery *et al*, 2001). High-grade dysplasia (HGD) confers a high probability of cancer, with rates varying between 31 and 59% over 5 years (Reid *et al*, 2000a; Buttar *et al*, 2001; Overholt *et al*, 2007). In recent years, there has been a paradigm shift in the treatment of HGD in BE from oesophagectomy to endoscopic therapy, with focal ablation of nodular disease (endoscopic mucosal resection (EMR)) and field ablation of residual flat dysplasia (photodynamic therapy (PDT), argon plasma coagulation, radiofrequency ablation and cryotherapy). The effectiveness of these approaches in eradicating HGD and reducing the risk of progression to cancer has been shown in randomised controlled trials (Overholt *et al*, 2007; Shaheen *et al*, 2009).

Although complete ablation of a Barrett's segment is the ideal response to treatment, complete reversal of HGD at 1 year is currently used as a marker of treatment success post-ablative therapy (Overholt *et al*, 2007; Shaheen *et al*, 2009). Nevertheless, in one series, late relapse beyond 2 years occurred in up to 23% of patients (Overholt *et al*, 2007). It is, therefore, necessary to perform regular surveillance endoscopy and biopsy, which is both unpleasant for patients and expensive. This has generated interest in the potential usage of biomarkers to predict success of treatment.

DNA ploidy abnormalities (aneuploidy/tetraploidy) measured by flow cytometry (FC) have been shown to be an independent risk factor for the development of cancer in untreated BE, independent of histology grade (Reid *et al*, 2000b; Rabinovitch *et al*, 2001). If a patient had both HGD and aneuploidy or DNA tetraploidy, the risk of developing cancer within 5 years was 66%, compared with 42% with HGD alone and 28% with DNA ploidy abnormalities alone. None of the 215 patients without HGD who were diploid (no cytometric abnormality) developed cancer during 5 years of follow-up (Reid *et al*, 2000b).

Image cytometric DNA analysis (ICDA) is a comparable technique to FC for the detection of DNA ploidy abnormalities; ICDA is advantageous as set-up cost is low, only a small number of nuclei are required, it is more sensitive for the analysis of tetraploid cell populations (Russack, 1994), and it is routinely performed on formalin-fixed paraffin-embedded (FFPE) samples, which allows analysis of archival material, valuable when planning longitudinal studies on disease progression.

The aim of this study is to evaluate the accuracy of ICDA *vs* FC, and to determine whether residual DNA ploidy abnormalities after successful treatment with PDT predict late relapse to HGD or cancer.

## Materials and methods

### Samples for comparative study of image cytometry and FC

A total of 35 patients from the UCLH BE database who underwent either oesophagectomy or EMR between 2005 and 2008 were randomly selected. A total of 48 FFPE blocks were retrieved and chosen for transportation to UW (16 EMR specimens and 32 blocks from oesophagectomy specimens). The EMR blocks were either low-grade dysplasia, HGD, carcinoma *in situ* or intramucosal cancer. Of the oesophagectomy specimens, 25 had invasive adenocarcinoma. A further seven blocks of cancer-free margins (six squamous oesophagus, one BE) were used as controls.

### Image cytometry

#### Preparation of the monolayer

Two 40 *μ*m sections were cut from FFPE tissue and transported at room temperature to UW, Seattle. The sample was then processed by a variation of the technique originally reported by Hedley *et al* (1983). Briefly the sections were dewaxed in xylene, gradually rehydrated in a step series of ethanol solutions, and digested using proteinase XXIV (Sigma-Aldrich, Dorset, UK) 2.5 mg for 2 h at 37°C. The sample was washed in phosphate-buffered saline (PBS), filtered though 40 *μ*m nylon mesh cell strainer (BD Biosciences, California, USA) and resuspended in 1.5 ml PBS. The nuclear suspension was then split with 500 *μ*l for ICDA and 1000 *μ*l for FC.

A total of 100 *μ*l of nuclear suspension was pipetted into a Shandon single use ez-cytofunnel (Thermo Scientific, Basingstoke, UK) and spun onto Superfrost Plus (blue) microscope slide (electrostatically permanently positive charge, VWR, Dorset, UK) using a Shandon Cytospin 2 (at × 225 **g** for 5 min) to form a nuclear monolayer. The monolayer was dried for 1 h and then placed in 200 ml HCl 5 mol l^–1^ for 1 h. The slide was then stained with Feulgen–Schiff reagent using standardised methodology (Bocking *et al*, 1995).

#### Measurement of DNA ploidy

The Fairfield DNA Ploidy system (Fairfield Imaging, Kent, UK) is an automated image cytometric analyser that consists of a Zeiss Axioplan microscope (Zeiss, Jena, Germany), a 546-nm green filter and a black-and-white, high-resolution digital camera (model C4742-95, Hamamatsu Photonics, Japan). Optical density and nuclear area were measured and integrated optical density of each nucleus was calculated. Background optical density was corrected for each nucleus. Segmentation software (a range of pre-defined criteria relating to the physical properties of the nuclei) automatically selects whole nuclei. At least 1000 nuclei were scanned automatically and sorted into four separate galleries for each cell type: nuclei of interest for measurement, lymphocytes, plasma cells and fibroblasts. The lymphocytes were used as reference cells to determine the position of the diploid peak (2c). The galleries were then edited manually to discard any cut or overlapping nuclei. The integrated optical density of each nucleus of interest was calculated and a histogram of DNA content produced. Ploidy-related parameters such as DNA index (DI) and percentages of cells exceeding 5c (5c ER) and 9c (9c ER) were also noted.

Histograms were analysed according to European Society for Analytical Cellular Pathology guidelines (Bocking *et al*, 1995) as follows:

A specimen was defined as diploid when there was only one peak (which was 2c, or DI=0.9–1.1) during the G0 or G1 phase, when the number of 4c nuclei during the peak of the G2 phase did not exceed 6% of the total, or when the number of nuclei with a DNA content of >5c did not exceed 1% of the total.
A specimen was defined as DNA tetraploid when there was a population of 4C nuclei (DI=1.9–2.1) >6% of the total, representing stage G2 of the cell cycle. The term ‘DNA tetraploid’ generally means a DNA content indistinguishable from that of tetraploid cells, with a percentage of these cells disproportionately higher than that of the S phase fraction.
A specimen was defined as aneuploid when there was a population of nuclei with abnormal DNA content, separated from the diploid peak (DI>1.1), and representing >2.5% of the total or when the number of nuclei with a DNA content of >5c or 9c exceeded 1% of the total. Aneuploid cases were further divided into near-diploid aneuploid (1.1–1.29) and aneuploid (1.30–1.89) (Lindahl *et al*, 1994).

All specimens were given unique coded identifiers and the histograms were reported blindly by two of three independent observers (JD, GM and MN). Consensus was reached in all cases.

### Flow cytometry

Standard FC was performed according to a conventional protocol and the manufacturer's instruction. Briefly, after splitting of the nuclear suspension, the 1000 *μ*l of supernatant was triturated with a 26 gauge needle, resuspended in an isotonic pH 7.4-buffered solution with 0.1% nonidet P-40 detergent, 10 *μ*g ml^–1^ diamidino-2-phenylindole and 1% RNAse, and filtered through 40 mm steel mesh. The analysis was performed on a Cytopeia InFlux cytometer using UV excitation. Chicken erythroid nuclei were used as reference cells to determine the position of the diploid peak (2c). A total of 50 000 cells were analysed, if available, and in all cases, acceptable histograms contained at least 10 000 cells and a coefficient of variation (CV) below 6.0%. The DNA content and cell cycle were analysed as earlier described using the software program MultiCycle (Phoenix Flow Systems, San Diego, CA, USA) (Rabinovitch, 1994).

### PDT patients

The criteria for inclusion were as follows:

- *Confirmed HGD before treatment*: At least two endoscopies before PDT with large-capacity four-quadrant biopsies every 2 cm of BE showing HGD. Histology was confirmed by two experienced independent specialist GI pathologists. The EMR was undertaken of any raised areas and only patients with residual HGD after EMR were given PDT. DNA ploidy was analysed on all four biopsies from each 2 cm level of BE. All patients had DNA ploidy analysis on HGD biopsies at one or multiple levels from their enrolment endoscopy.
- *Successful treatment with no dysplasia at follow-up*: All patients were treated with five aminolaevulinic acid (ALA) PDT as described earlier (Mackenzie *et al*, 2007, 2009). Ethical approval was granted for the study (EudraCT No: MF 8000 21074).

After PDT patients underwent endoscopy with four-quadrant biopsies every 2 cm from the treated oesophagus at 6 weeks, 4 and 12 months after PDT and at 18, 24, 36, 48 and 60 months. At each follow-up, endoscopy after PDT care was taken to ensure that the whole of the treated area was sampled, to ensure no buried glands were missed in areas that had healed with squamous regeneration. Assessment of DNA ploidy post-PDT was carried out on all specimens with glandular epithelium, or mixed squamo-glandular epithelium. Biopsies with squamous epithelium alone were not analysed.

All patients treated with PDT who were free of residual disease at 12 months were included in this prospective study. To be considered disease free, patients must have had at least three endoscopies over at least 12 months post-PDT. Relapse was defined as presence of HGD or cancer during follow-up.

### Statistical methods

All analysis was performed using either SPSS for Windows statistical package (SPSS Inc., Version 14.0, Chicago, IL, USA) or Stata for Windows (StataCorp LP, Version 10.1, College Station, TX, USA) The two-tailed *P-*value of <0.05 was considered significant. The differences between FC and IC of the CV, mean nuclei of G1 peak and DI of aneuploid peak were analysed using paired *t*-tests.

Hazard ratios (HRs) for late relapse (HGD or cancer beyond 1 year) were estimated using the Cox proportional hazards model and their significance was assessed using the log-rank test (for categorical factors: sex, the presence of DNA ploidy abnormalities before 4 and 12 months after treatment) or the Wald's test (for continuous variables: age and length of BE pre-PDT).

## Results

### Comparison of FC *vs* ICDA

A total of 44 samples from 31 patients were successfully analysed, and 93% (41 out of 44) were classified identically between the two centres. All seven controls were diploid at both centres. Of the 34 cases for which there was agreement, 67% were aneuploid, 9% tetraploid and 24% diploid. Coefficient of variation of the G1 peak was lower by FC than ICDA (*P*=0.04). Mean number of nuclei collected was significantly lower by ICDA (520) than FC (12050) (*P*<0.001). There was no significant difference in the DI of the aneuploid peak between the two methods. A summary of the data is presented in Appendix (Table A1.

**Figure 1:**
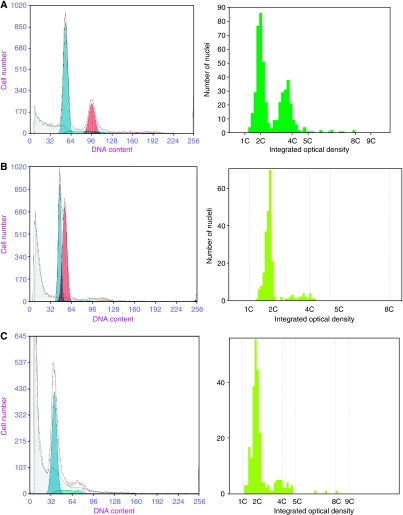
Histograms by FC and ICDA (left to right). (**A**) Aneuploid case by FC and ICDA. (**B**) Discordant case, near-diploid aneuploid by FC and diploid by ICDA. Note that both analysis methods show evidence of separate G_2_ peaks. (**C**) Discordant case, diploid by FC and tetraploid by ICDA.

Representative histograms are shown in Figure 1. Three cases gave discordant results. One was reported as near-diploid aneuploid by FC, but diploid by ICDA. The near-diploid peak at DI=1.16 diagnosed by FC (Figure 1B) is not found in image cytometry galleries, although two small blips in the S phase that are likely to be the G2 of each peak are apparent in both FC and ICDA histograms. One explanation is that because of poor staining, the real G1 peak was gated out by ICDA software, which is confounded by the low nuclei number analysed (*n*=236) when compared with FC (*n*=13942). This case shows the strength of FC, as analysis of larger cell samples provides histograms with better resolution and, therefore, aneuploid peaks in the near-diploid range are more readily detected.

A second discordant case (not shown) had similar histograms between the two centres, but a small aneuploid peak of 5.9% of the total, seen at DI=1.44 by FC, was not of sufficient magnitude to be called aneuploid by ICDA.

The third discordant case (Figure 1C) was classified as DNA tetraploid by ICDA, but diploid by FC (Figure 1C). There were low nuclei numbers for both centres in this sample, though when analysing the FC histogram, there is a high level of background aggregates and debris, which overlies the 4N peak and complicates its quantitation. ICDA allowed inspection of the events within this peak and abnormal nuclei were shown within the image gallery at the 4c region. This case shows an advantage of ICDA over FC, the ability to directly visualise abnormal nuclei and make a diagnosis of DNA ploidy abnormalities even if debris or aggregates overlap with the 4c region.

### ICDA post-PDT

A cohort of 30 patients who were treated with ALA PDT for HGD arising in BE, and who remained clear of dysplasia for at least 12 months after treatment, were studied for factors predictive of future relapse. In particular, DNA ploidy was assessed by ICDA on all biopsies taken before treatment, and at 4 and 12 months post-treatment. There were 13 cases of late relapse (7 cancer, 6 HGD) and 17 patients who remained free of relapse for a mean of 44 months (including 12 months).

There were 1177 months of follow-up (from treatment) in the 30 patients (mean 39 months/patient, IQR 12–64 months) corresponding to just over 68 years beyond 1-year post-treatment. The annual rate of recurrence (including cancer beyond 1 year) was 19%. Median relapse-free interval was 3.6 years.

Patient characteristics and results of survival analysis are shown in Table 1. None of age, sex or length of Barrett's pre-PDT had a significant effect on late relapse.

**Figure 2:**
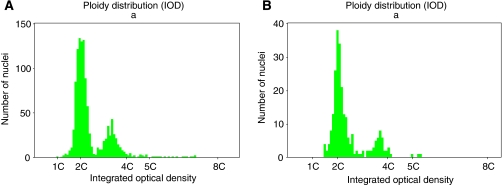
Histograms from a patient who relapsed to cancer at 24 months. (**A**) Aneuploid histogram before PDT with DI=1.7. (**B**) Persistent aneuploidy post-PDT with DI=1.7.

**Figure 3:**
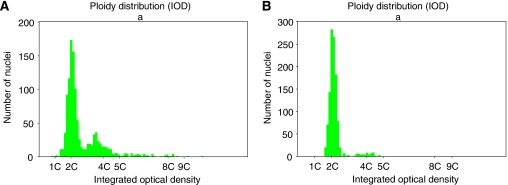
Histograms from a patient who was disease free at 42 months. (**A**) Aneuploid histogram before PDT with DI=1.8. (**B**) Diploid 4 months post-PDT.

A total of 469 samples were processed for DNA ploidy from these 30 patients. A summary of the raw data is presented in Appendix (Tables A2 and A3). Representative histograms are shown in Figures 2 and 3.

Before treatment, all but 5 of the 30 patients were aneuploid and there was no significant difference in relapse rates with aneuploidy. At 4 months after treatment, 10 patients were aneuploid. Patients who were aneuploid at 4 months were significantly more likely to have late relapse (HR=4.1, *P*=0.009). At 12 months DNA ploidy was assessed in 29 patients: 10 were aneuploid and 2 had DNA tetraploidy, both also had aneuploidy. Aneuploidy at 12 months was a significant predictor of subsequent relapse (HR=3.6, *P*=0.03).

**Figure 4:**
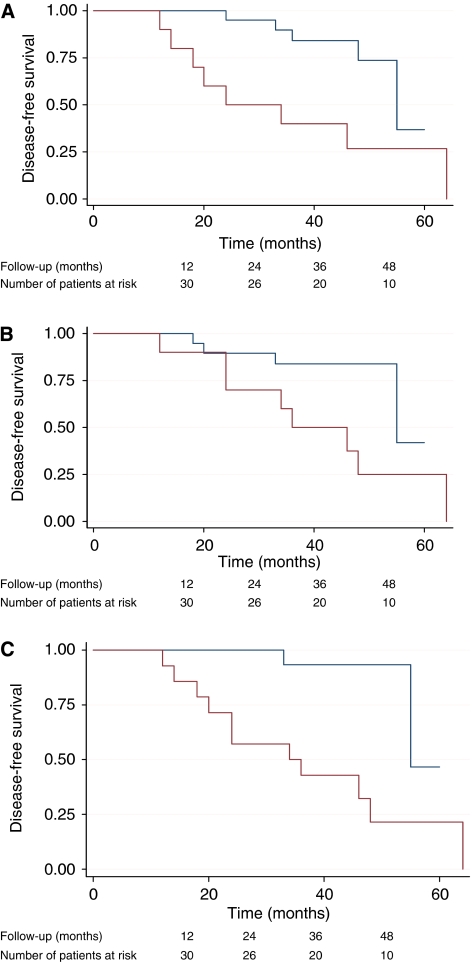
Kaplan–Meier disease-free survival estimates according to DNA ploidy status at (**A**) 4 months, (**B**) 1 year and (**C**) at both time points post-ALA PDT. Blue=diploid post-PDT, red=DNA ploidy abnormalities post-PDT (The colour reproduction of this figure is available on the html full text version of the manuscript).

We then compared all 14 patients with DNA ploidy abnormalities at 4 or 12 months with those with that were diploid. This variable was extremely predictive of recurrence beyond 12 months: HR=8.2 (1.8–37.8) log-rank *P*=0.001 (Figure 4C). It is noticeable that the survival curves begin to diverge earlier for DNA ploidy at 4 months compared with DNA ploidy at 12 months (Figure 4A and B). We, therefore, also looked at DNA ploidy as a time-dependent covariate, taking DNA ploidy at 4 months as the covariate value for recurrence between 12 and 20 months and DNA ploidy at 12 months (if available) to predict recurrence beyond 20 months post-treatment. This time-dependent covariate gave an HR of 6.3 (1.7–23.4), log-rank *P*=0.0015. None of the other variables recorded had a significant effect on recurrence beyond 12 months.

## Discussion

These data show that ICDA is highly accurate for the diagnosis of DNA ploidy abnormalities when compared with FC, the current gold standard. These findings are strengthened by the blinded study design, and comparison with a reference laboratory with a wealth of experience in FC and BE. This experiment shows the potential advantages of each technique. ICDA accurately quantifies DNA tetraploidy, by permitting direct visualisation and selection of cell populations that are in the 4c region. The importance of DNA tetraploidy in BE has been earlier documented by the Seattle group, with a tetraploid fraction above 6% associated with an elevated risk of progression to cancer (Rabinovitch *et al*, 2001). Moreover, it is unusual to find an elevated S phase fraction in combination with a DNA tetraploid fraction above 6%, consistent with the group's published findings that elevated S phase fractions are not statistically associated with risk of progression to cancer in BE, whereas DNA tetraploid fractions are.

In contrast to ICDA, FC analysed significantly larger cell samples and provided histograms with better resolution, so aneuploid peaks in the near-diploid range were more readily detected. The importance of near-diploid aneuploidy is unclear. Earlier FC data from UW showed that 9% of the patients with DI=1.1–1.35 progressed to cancer, compared with 44% who had DI >1.35 (Rabinovitch *et al*, 2001). Importantly, in that study, no patient with a near-diploid DNA content progressed to cancer within 5 years of their baseline endoscopy.

The comparison of the two techniques has been evaluated in many tumours with concordance rates of 70–94% in breast cancer (Lee *et al*, 1991; Baldetorp *et al*, 1992; Chen *et al*, 1995) and 81–100% in gynaecological tumours (Kaern *et al*, 1992; Esposito and Fuchs, 1994). In BE, the published data comparing the two techniques are limited to a single study by Goyal *et al* on 27 patients, that is 10 normal controls and 17 with Barrett's adenocarcinoma (Huang *et al*, 2008). Image cytometry was carried out on thin sections of FFPE tissue and areas of interest marked before scanning using Automated Cellular Imaging System. This method is potentially advantageous as mixed squamo-glandular epithelium, which may variably dilute the glandular epithelium of interest, is not included. The authors concluded that IC detected aneuploidy in all adenocarcinoma samples, whereas FC missed the diagnosis of aneuploidy in 29%. There were, however, limitations to this study. IC and FC were carried out in the same laboratory with no independent review of histograms, and there was no validation of FC technique. The two techniques were carried out on different cut samples (7 *μ*m IC, 2 × 50 *μ*m FC), thereby making it difficult to draw a direct comparison. Finally, no patients were reported as DNA tetraploidy, an important independent marker of disease progression. This may be explained by the study design, as only cancers were analysed for DNA ploidy, and DNA tetraploidy can appear early in the cascade of genetic change. This may also be explained by introduction of a cutting error of the larger tetraploid nuclei when using 7 *μ*m sections, leading to underestimation of tetraploid fraction.

We have gone on to show the value of DNA ploidy measured by ICDA as a biomarker to predict late relapse to HGD and cancer in BE, after successful treatment of dysplasia by PDT. DNA ploidy abnormalities after treatment conferred an HR of 8.2 (1.8–37.8) for developing recurrent HGD or cancer. Rabinovitch *et al* (2001) earlier showed that aneuploidy arising in non-dysplastic BE, as measured by FC, conferred a relative risk for progression to cancer of 4.4 (CI=1.4–14). These earlier data are consistent with the findings of our study.

The importance of residual genetic abnormalities in Barrett's epithelium after PDT has earlier been postulated. Foultier *et al* (1994) assessed the influence of DNA ploidy abnormalities on outcomes in patients with early gastrointestinal cancer (oesophageal, gastric, colorectal) treated by haematoporphyrin derivative PDT. Aneuploidy (measured by FC) at 4-month follow-up was associated with a poor response, with only 5 of 15 patients with aneuploidy achieving complete remission, compared with 12 of 17 patients without aneuploidy. Prasad *et al* (2008) reported on the correlation of histology with biomarker status after photofrin PDT, in which overall fluorescence *in situ* hybridisation positivity for a panel of biomarkers (including loss of p16 and p53) was seen in 60% of non-responders to sp-PDT *vs* 19% of responders. No individual biomarker, however, was shown to predict success or failure of therapy.

DNA ploidy changes before and after PDT suggest that ALA acts directly on cancer cells in addition to vascular effects leading to oxygen deprivation and apoptosis (Foultier *et al*, 1994). A total of 13 out of 30 patients did not achieve reversal of DNA ploidy abnormalities despite normal histology, which suggests that dysplastic cell populations in BE do not act in the same way to treatment. One postulated theory on the histogenesis of BE is that heterogeneity arises from multiple independent clones, in contrast to the selective sweep to fixation model of clonal expansion described earlier (Leedham *et al*, 2008). This may explain why sub-populations of cells may occur that are resistant to therapy and continue to display genetic abnormalities.

The occurrence of diploid cell populations in HGD tissue was noted in five patients before treatment. A total of 3 out of 5 patients have relapsed, with 2 out of 3 cases showing no DNA ploidy abnormalities in the first year. Further analysis of DNA ploidy was undertaken on all biopsies taken from these patients after 1 year, and both showed DNA ploidy abnormalities before developing cancer. In the first, aneuploidy was found at 2 years post-PDT, 6 months before cancer was diagnosed. In the second, DNA tetraploidy was found 18 months post-PDT. Cancer developed 3 years later. This may be explained by sampling error, as small aneuploid populations may be missed by ICDA. Another argument would be ALA PDT induced mutation and up-regulation of an aneuploid clone not earlier quantified. This has been shown in studies on glioblastoma cell lines in which ALA can lead to up-regulation of putative cancer stem cells that are resistant to therapy (Morgan and Petrucci, 2009).

There are some limitations to this study. Despite significant differences between groups, the sample size is small. Kaplan–Meier plots and survival analysis methods allow for whatever follow-up time is available for each person, thereby maximising our sample size and allowing statistically significant conclusions to be drawn. The significance of endoscopic sampling error is difficult to quantify. It is possible that small foci of dysplasia may be missed at follow-up endoscopy using current standard four-quadrant surveillance. However, after three clear endoscopies, we estimate this miss rate to be small.

DNA ploidy measured by ICDA is accurate when compared with FC, with advantages of cost effectiveness, potential for automation and routine analysis of paraffin-embedded tissue. It is important to note that both ICDA and FC examine predominately whole nuclei, and it is less likely that these findings would be replicated by analysis of cut nuclei from thin sections. Furthermore, DNA ploidy abnormalities by ICDA predict risk of relapse after ablative therapy, and may be clinically useful as a biomarker by allowing an individualised approach to patient follow-up. If stratifying risk according to DNA ploidy status after ablative therapy, then patients with residual aneuploidy would require intensive surveillance, whereas diploid patients may return to 3 yearly surveillance and be reassured of a very low cancer risk. The reduction in the frequency of follow-up endoscopies for the majority of patients would also provide financial savings for healthcare services. The study is limited by its retrospective design and a larger prospective study using reversal of DNA ploidy abnormalities as an end point for treatment success would be valuable.

## Figures and Tables

**Table A1:**
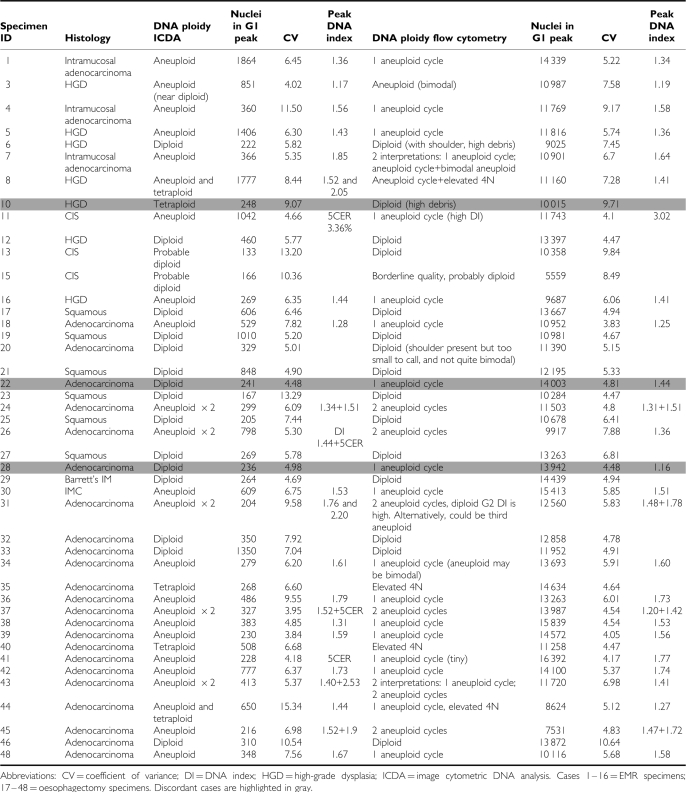
Comparison of flow cytometry *vs* image cytometric DNA analysis (ICDA)

**Table 1:** Patient characteristics and survival analysis

	**Mean**	**IQR**	**HR**	**95%CI**	***P*-value**
Male	83%		0.47	0.12–1.78	0.25
Age (years)	69	60–77	1.01	0.96–1.07	0.62
Barrett's length before PDT (cm)	5.7	4–7	1.07	0.88–1.30	0.51
Aneuploidy pre-PDT	83%		0.75	0.20–2.79	0.66
Aneuploidy 4 months post-PDT	33%		4.1	1.3–13.0	0.009
Aneuploidy 12 months post-PDT (*n*=29)	34%		3.6	1.05–12.3	0.029
Aneuploidy 4 and/or 12 months post-PDT	47%		8.2	1.8–37.8	0.0012

Abbreviations: CI=confidence interval; HR=hazard ratio; PDT=photodynamic therapy.

**Table A2:** Cases of progression to HGD or adenocarcinoma

**Study ID**	**Length BE (cm)**	**DNA ploidy status at baseline**	**No. of levels DNA ploidy**	**No. of levels of HGD**	**Peak DI**	**Mean CV**	**Mean *n* in G1 peak**	**Peak 5c**	**DNA ploidy status at 4 months**	**No. of levels DNA ploidy**	**Histology**	**Peak DI**	**Mean CV**	**Mean *n* in G1 peak**	**Peak 5c**	**DNA ploidy status at 12 months**	**No. of levels DNA ploidy**	**Histology**	**Peak DI**	**Mean CV**	**Mean *n* in G1 peak**	**Peak 5c**
1	6	Aneuploid	1	1	1.51	7.2	744	2.13	Hypodiploid	1	IM	0.73	8	777	0	Diploid	0	LGD	1.01	6.8	852	0
2	6	Aneuploid and tetraploid	1	1	1.32/2.02	10.9	1018	0.54	Aneuploid	2	Indefinite	1.52	9.2	557	0.74	Aneuploid	1	IM	1.26	10.5	985	0.17
3	7	Aneuploid, tetraploid	2	1	1.76/1.97	7.7	863	0.99	Aneuploid and tetraploid	2	IM	1.29/1.99	10.7	363	0.23	Aneuploid and tetraploid	1	LGD	0.5/2.05	6.8	576	0
4	3	Aneuploid	1	1	1.89	9.6	907	1.20	Aneuploid	1	Glandular mucosa	1.65	10.2	807	0.41	Aneuploid	1	IM	1.88	9.5	510	0.4
5	11	Aneuploid and tetraploid	3	2	1.48/1.97	6.7	503	1.07	Aneuploid	2	Indefinite	1.75	6.0	681	0.29	Not reportable		IM				
6	3	Aneuploid	1	1	1.89	15.1	986	0.76	Aneuploid	2	IM	1.63	9.0	525	0.14	Aneuploid	1	Indefinite	1.38	5.0	923	0.15
7	5	Aneuploid	2	1	1.56	9.3	725	0.37	Diploid	0	Indefinite	1.00	4.4	1103	0	Hypodiploid and tetraploid	1	IM	0.50/1.97	10.1	392	0
8	4	Aneuploid	1	1	1.83	10.4	648	0	Diploid	0	Glandular mucosa	1.01	5.9	916	0	Aneuploid	3	Glandular mucosa	1.85	11.6	224	1.50
9	13	Aneuploid and tetraploid	6	3	1.74/2.05	9.43	708	1.79	Aneuploid	1	IM	1.79	6.3	680	0.11	Aneuploid	1	IM	1.87	7.4	773	0.26
10	3	Aneuploid	1	1	1.31	8.6	626	0.78	Aneuploid	3	Mixed sqaumo-glandular	1.83	6.4	745	0.21	Diploid	0	Mixed squamo-glandular	1.00	4.6	1051	0
11	5	Diploid	0	1	0.99	5.7	888	0	Diploid	0	Glandular mucosa	1.0	4.2	903	0	Aneuploid	1	Glandular mucosa	1.40	13	399	0.83
12	4	Diploid	0	1	1.01	6.7	1194	0	Diploid	0	IM	1.02	6.3	1064	0	Diploid	0	IM	1.03	11.1	809	0
13	2	Diploid	0	1	1.03	9.4	1274	0	Diploid	0	Indefinite	0.99	7.1	1059	0	Diploid	0	Indefinite	1.01	8.5	945	0

Abbreviations: BE=Barrett's oesophagus; CV=coefficient of variance; DI=DNA index; HGD=high-grade dysplasia; ICDA=image cytometric DNA analysis; LGD=low-grade dysplasia.

Corresponding histology and raw ICDA data at three time points are shown.

**Table A3:** Controls with no relapse

**Study ID**	**Length BE (cm)**	**DNA ploidy status at baseline**	**No. of levels DNA ploidy**	**No. of levels of HGD**	**Peak DI**	**Mean CV**	**Mean *n* in G1 peak**	**Peak 5c**	**DNA ploidy status at 4 months**	**No. of levels DNA ploidy**	**Histology**	**Peak DI at 4/12**	**Mean CV**	**Mean *n* in G1 peak**	**Peak 5c**	**DNA ploidy status at 12 months**	**No. of levels DNA ploidy**	**Histology**	**Peak DI**	**Mean CV**	**Mean *n* in G1 peak**	**Peak 5c**
1	8	Aneuploid	3	2	1.66	10.1	695	0.33	Aneuploid	2	IM	1.61	11.1	650	0.12	Aneuploid	2	IM	1.56	7.8	288	0.63
2	5	Aneuploid	3	1	1.61	6.6	419	0.69	Aneuploid	2	Glandular mucosa	1.64	12.2	572	0.40	Diploid	0	Glandular mucosa	1.03	10.0	223	0
3	11	Aneuploid	3	1	1.81	11.5	737	0.90	Diploid	0	IM	1.01	7.0	1040	0	Diploid	0	IM	1.02	4.0	852	0
4	5	Aneuploid	1	1	1.27	6.8	783	0.30	Diploid	0	Indefinite	1.01	7.8	433	0	Diploid	0	IM	1.00	8.2	425	0
5	4	Aneuploid	2	1	1.77	10.5	448	0.23	Diploid	0	Glandular mucosa	1.01	7.0	1184	0	Diploid	0	Glandular mucosa	1.01	4.5	925	0
6	7	Aneuploid	2	2	1.81	7.2	661	2.57	Diploid	0	Glandular mucosa	1.01	7.9	708	0	Diploid	0	IM	1.01	6.8	455	0
7	6	Aneuploid	1	1	1.21	7.8	761	0.09	Diploid	0	IM	1.03	4.8	991	0	Diploid	0	IM	0.99	4.3	850	0
8	5	Aneuploid	1	1	1.45	9.7	881	0	Diploid	0	LGD	1.02	8.6	1066	0	Diploid	0	LGD	1.03	4.0	888	0
9	3	Aneuploid	1	1	1.81	8.5	1071	0.09	Diploid	0	IM	1.03	6.2	953	0	Diploid	0	IM	1.03	9.9	885	0
10	4	Aneuploid	2	2	1.34	6.2	338	2.30	Diploid	0	Glandular mucosa	1.01	4.3	1051	0	Diploid	0	IM	1.01	9.2	402	0
11	5	Aneuploid	1	1	1.83	6.5	1152	0.1	Diploid	0	IM	1.04	8.8	1197	0	Diploid	0	Glandular mucosa	1.02	9.1	355	0
12	4	Aneuploid	2	1	1.89	7.0	921	1.49	Diploid	0	Glandular mucosa	1.00	7.5	482	0	Diploid	0	Mixed squamo-glandular	1.00	7.9	1200	0
13	4	Aneuploid	2	1	1.89	7.1	423	4.04	Diploid	0	Glandular mucosa	1.00	5.7	1000	0	Diploid	0	Glandular mucosa	0.99	5.8	859	0
14	4	Aneuploid	1	1	1.85	11.2	905	2.68	Diploid	0	Glandular mucosa	0.99	6.7	923	0	Diploid	0	Glandular mucosa	0.99	5.2	890	0
15	8	Aneuploid	1	1	1.56	8.3	587	0.74	Diploid	0	IM	1.00	4.1	1182	0	Diploid	0	IM	1.01	5.8	903	0
16	6	Diploid	0	1	1.02	4.9	769	0.20	Diploid	0	Mixed squamo-glandular	1.01	9.7	1098	0	Aneuploid	4	Mixed squamo-glandular	1.85	7.9	235	0.12
17	3	Diploid	0	1	1.01	6.0	408	0	Diploid	0	Glandular mucosa	1.01	6.6	752	0	Diploid	0	Glandular mucosa	1.04	10.3	1175	0

Abbreviations: BE=Barrett's oesophagus; CV=coefficient of variance; DI=DNA index; HGD=high-grade dysplasia; ICDA=image cytometric DNA analysis; LGD=low-grade dysplasia.

Corresponding histology and raw ICDA data at three time points are shown.

## Appendix A1

**Raw data**

Table A1

Table A2

Table A3
